# Sex Differences in the Prevalence and Correlates of Sleep Disturbance Among Indonesian Stroke Survivors: A Population-Based Study of 7,147 Individuals

**DOI:** 10.12688/f1000research.182846.4

**Published:** 2026-09-15

**Authors:** Sri Susanty, Renny Wulan Apriliyasari, Hsiao-Yean Chiu, Rusdi Bin ABD Rashid, Era Catur Prasetya, Kanjanee Phanphairoj, Mokh. Sujarwadi, Faizul Hasan

**Affiliations:** 1 Faculty of Nursing, Chulalongkorn University, Bangkok, Bangkok, Thailand; 2 Nurse Professional Education Study Program, Faculty of Medicine, Universitas Halu Oleo, Kendari, South East Sulawesi, Indonesia; 3 School of Nursing, Taipei Medical University, Taipei City, Taipei City, Taiwan; 4 Department of Psychological Medicine, University of Malaya Faculty of Medicine, Kuala Lumpur, Federal Territory of Kuala Lumpur, Malaysia; 5 Faculty of Medicine, Universitas Muhammadiyah Surabaya, Surabaya, East Java, Indonesia; 6 Faculty of Nursing, Universitas Jember, Jember, East Java, Indonesia

**Keywords:** sleep, post-stroke insomnia, stroke, insomnia, Asian.

## Abstract

**Objectives:**

Sleep disturbance is a prevalent issue among those who have experienced a stroke. The impact of gender on the prevalence of sleep disturbance within Asian stroke populations remains ambiguous. This research seeks to examine the prevalence of sleep disturbance among stroke survivors in Indonesia.

**Methods:**

This quantitative prevalence analysis was based on the 2018 Indonesian Basic Health Research. A total of 7,147 stroke survivors who answered the sleep disturbance questions were included. The estimated prevalence and regression models were examined utilizing SPSS software.

**Results:**

The total prevalence of sleep disturbance among stroke survivors in Indonesia was 28.4% (95% CI: 27.4%–29.4%). Females exhibited a greater frequency of sleep disturbance than males (34.4% versus 26.1%, respectively). When categorized by age group, both females and males exhibited a trend of rising sleep disturbance prevalence with time. Moreover, daily smoking, low educational attainment, rural residency, and insufficient physical activity were correlated with sleep disturbance symptoms in the male cohort. In females, the presence of comorbidities such as cardiovascular disease and asthma was associated with sleep disturbance.

**Conclusions:**

This study’s findings revealed a significant prevalence of sleep disturbance among stroke survivors in Indonesia. Our findings indicate a gender influence on sleep disturbance prevalence, with females exhibiting a higher prevalence than males. These findings underscore the significance of sleep assessment and treatment in clinical or community environments.

## 1. Introduction

Sleep disturbance is a common problem around the world. The prevalence rate ranges from 35% to 43% from the acute to the chronic phase. ^1^ ^–^ ^3^ Stroke patients with sleep disturbance have a higher risk of developing cognitive impairment ^4^ and reduced health-related quality of life. ^5^ ^,^ ^6^ Researchers and clinicians are now paying high attention to managing the sleep disturbance problem.

Previously, two studies investigating the global prevalence of sleep disturbance using a meta-analytic design have been published. ^1^ ^,^ ^2^ One meta-analysis, involving 22 studies on sleep disturbance, found the prevalence to be 38.2%. ^1^ The updated meta-analysis, which included 28 studies, suggested a prevalence rate of 39.7%. ^2^ However, most of the included studies were derived from Western populations. Adding evidence by investigating the sleep disturbance prevalence among Eastern populations is important.

As a note, gender plays a critical role in the prevalence of sleep disturbance. ^7^ Previous meta-analyses suggest that females are more prevalent than males in terms of sleep disturbance in the general population in China. ^8^ ^,^ ^9^ However, the effect of gender on sleep disturbance among stroke survivors remains under research. Moreover, the available studies involved a small sample size. ^1^ ^,^ ^2^ Hence, there is a need to conduct an updated study investigating the effect of gender on sleep disturbance using a larger sample size. This study aims to investigate the effect of gender on the prevalence of sleep disturbance among stroke survivors using multicenter nationwide data.

## 2. Materials and methods

### 2.1 Study sample

The data utilized in this study were obtained from the 2018 Basic Health Research (Riset Kesehatan Dasar, RISKESDAS) conducted by the Indonesian Ministry of Health. The RISKESDAS survey is a thorough national evaluation conducted quinquennially to collect health-related data. The National Institute of Health Research and Development (NIHRD) administers it under the Ministry of Health in Indonesia. The survey was administered by enumerators possessing formal education in health, holding at least health diplomas. The enumerators performed face-to-face interviews utilizing validated questions for accuracy and dependability. Furthermore, they performed measurements and assessments during home visits, as outlined in the supplementary document issued by the Ministry of Health in Bahasa Indonesia (Kementerian Kesehatan, 2019). Members of each household (those residing on the premises for at least six months and sharing the same food income source) were solicited to partake in the survey. Health-related data on patients diagnosed with stroke was obtained from the 2018 RISKESDAS dataset.

### 2.2 Inclusion and exclusion criteria

We only considered participants who satisfied the requirements listed below in order to meet the inclusion criteria: 1) having received a stroke diagnosis in the past; and 2) having submitted the necessary information for the sleep disturbance variable. Participants whose sleep disturbance variable had missing values were not included in the study.

### 2.3 Dependent variable

A question related to sleep disturbance was examined: “In the past two weeks, have you experienced any sleep disturbances, such as difficulty initiating sleep, nocturnal awakenings, or early morning arousal?” The sleep disturbance status was assumed if participants answered ‘yes’. We acknowledge that this single-item measure assesses self-reported sleep disturbance and does not fulfill diagnostic criteria for clinical insomnia, as it does not assess duration, frequency, daytime impairment, adequate sleep opportunity, or differential diagnoses. Therefore, our findings represent self-reported sleep disturbance rather than clinically diagnosed insomnia.

### 2.4 Independent variables

2.4.1 Sociodemographic variables

Sociodemographic characteristics encompassed age, gender, educational attainment, marriage status, occupation, and degree of urbanization. We classified educational attainment into three categories: ‘no formal education’ (0), ‘non-university level’ (1), and ‘university level’ (2). Marital status was classified as ‘single’ (0) or ‘married’ (1), and ‘widow or widower’ (2). Occupation status was categorized as ‘unemployed’ (0) and ‘employed’ (1), and urbanization level as ‘rural’ (0) and ‘urban’ (1).

2.4.2 Health and lifestyle behavior variables

In this research, we incorporated health conditions and lifestyle behaviors, including regular follow-up, comorbidities (such as cancer, asthma, cardiovascular disease, and chronic renal illness), vigorous and moderate physical activity, smoking status, and alcohol consumption.

The evaluation of regular follow-up for stroke was conducted using the subsequent inquiry: Has your stroke state been consistently monitored by a physician at the healthcare facility? the potential responses of “yes,” ‘sometimes,’ and ‘no.’

Comorbidities were identified based on the response (‘yes’ = 0 or ‘no’ = 1) to the question: ‘Have you received a diagnosis of (….) from a physician?’

Smoking status was assessed by the inquiries: ‘Are you a smoker?’ and ‘Have you smoked in the past month?’ Smoking status was classified as ‘non-smoker’ (code = 0), ‘occasional smoker’ (code = 1), and ‘daily smoker’ (code = 2).

Alcohol intake was evaluated by the inquiry: ‘Have you ingested alcohol in the past month?’ with response options of ‘yes’ and ‘no.’

Physical activity was evaluated utilizing the modified Global Physical Activity Questionnaire (GPAQ) from the World Health Organization for surveillance purposes. The GPAQ gathers data on physical activity in three areas: occupational or domestic activities, transportation, and leisure pursuits. All activities must have been conducted uninterrupted for a minimum of 10 minutes per week. Participants were requested to enumerate activities they typically engaged in daily, categorizing them as ‘yes’ for activities performed and ‘no’ for those not performed, for both vigorous and moderate physical activity. The assessment of GPAQ demonstrated satisfactory inter-rater reliability for categorical variables, with kappa values between 0.44 and 0.78, and test-retest reliability for continuous variables, with Spearman’s rho ranging from 0.45 to 0.80. ^10^

### 2.5 Ethical consideration and informed consent statement

Ethical approval for the original RISKESDAS 2018 data collection was granted by the Ethical Committee of Health Research, NIHRD, Ministry of Health, Republic of Indonesia (No. LB.02.01/2/KE.267/2017). Secondary analysis for the present study was approved by the Health Research Ethics Committee of the Faculty of Medicine, Universitas Halu Oleo (No. 62/UN29.20.2/ETIK/2025). Written informed consent was obtained from all participants in the original survey. As this study uses only de-identified secondary data, no additional consent was required.

### 2.6 Statistical analysis

Statistical analysis was conducted utilizing SPSS (Version 29, SPSS Inc., Chicago, IL, USA). Descriptive statistics were analyzed for demographic features and aggregated responses, encompassing the mean and standard deviation for continuous variables and percentages for categorical data. Subsequently, we performed analyses utilizing χ2 (Chi-square) for categorical variables and the Mann-Whitney U-test (given the data’s deviation from a normal distribution) for continuous variables to investigate the presence of gender disparities in sociodemographic characteristics, health conditions, and lifestyle behaviors.

Acknowledging that RISKESDAS employs a complex survey design with clustering, stratification, and sampling weights, we note that our analysis did not incorporate these design features due to technical constraints. Consequently, our findings should be interpreted as estimates from a large sample rather than nationally representative point estimates.

**Figure 1.**
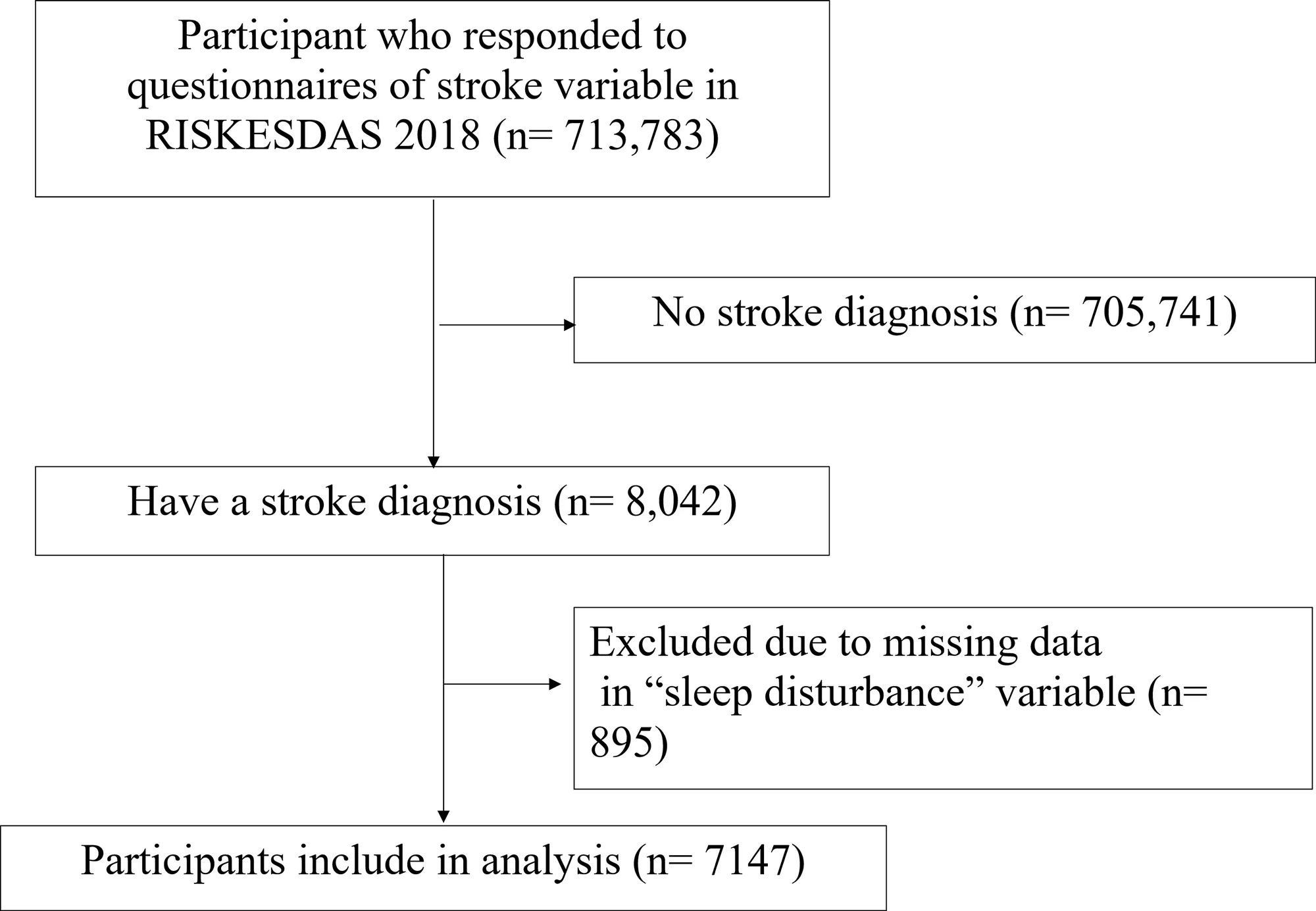
Participant flow chart. n = number of participants.

For the regression analyses, complete-case analysis was employed. Participants with missing sleep disturbance data (n = 895) were excluded, yielding the analytic sample of 7,147 participants ( Figure 1). For the regression models, no missing values were found for any independent variable; thus, all analyses included the full sample of 7,147 participants. Multivariable logistic regression was performed using forced entry (simultaneous) method, including all prespecified covariates based on clinical and theoretical relevance. We assessed multicollinearity using variance inflation factors (VIFs), and no significant collinearity was detected (all VIFs < 2.0). Given our primary interest in identifying correlates of sleep disturbance separately in males and females due to known biological and social differences, we conducted sex-stratified models. To formally test whether associations differed by sex, we performed interaction analyses (sex × independent variable). Hosmer–Lemeshow goodness-of-fit tests were performed to evaluate model calibration. The alpha criterion for hypothesis testing was established at p < 0.05.

## 3. Results

### 3.1 Study characteristic

At the beginning, 713,783 individuals were screened for eligibility. Among them, 705,741 participants were excluded because they did not have a stroke diagnosis. Furthermore, 895 participants were excluded because they had missing values on the sleep disturbance variable. The final analysis comprised 7,147 stroke participants ( Figure 1).

Table 1 presents a comparison of participants’ characteristics between males and females. The sleep disturbance cohort exhibited a greater mean age than the non-sleep disturbance cohort across both genders. Among males, educational attainment, work position, urbanization, consistent follow-up, and the presence of comorbidities such as cancer, heart disease, asthma, and participation in regular physical exercise exhibited significant differences between the sleep disturbance and non-sleep disturbance groups (all P < 0.05). Among females, educational attainment, marital status, and the occurrence of comorbidities such as cardiovascular disease and asthma exhibited significant differences between the sleep disturbance and non-sleep disturbance cohorts (all P < 0.05). Additional demographic information is available in Table 1 and Table 2.

**Table 1.** Demographic characteristic of participants.

			Male (n = 3574)				*p*-value	Female (n = 3573)				*p*-value
	Total		Sleep Disturbance		No Sleep Disturbance			Sleep Disturbance		No Sleep Disturbance		
Variables	n	(%)	n	(%)	n	(%)		n	(%)	n	(%)	
	7147	(100)	932	(26.1)	2642	(73.9)		1230	(34.4)	2343	(65.6)	
Age, mean (SD)	58.7	(12.5)	60.3	(12.2)	59.1	(11.9)	0.01	57.37	(11.93)	55.87	(12.24)	0.01
Smoking status, n (%)							0.48					0.86
Non-smoker	4611	(64.5)	294	(31.5)	949	(35.9)		1161	(94.4)	2207	(94.2)	
Yes (not every day)	760	(10.6)	179	(19.2)	475	(18)		34	(2.8)	72	(3.1)	
Yes (everyday)	1776	(24.8)	459	(49.2)	1218	(46.1)		35	(2.8)	64	(2.7)	
Education, n (%)							<0.001					0.02
No formal education	732	(10.2)	79	(8.5)	168	(6.4)		181	(14.7)	304	(13)	
Non-university level	5810	(81.3)	781	(83.8)	2145	(81.2)		995	(80.9)	1889	(80.6)	
University level	605	(8.5)	72	(7.7)	329	(12.5)		54	(4.4)	150	(6.4)	
Marital status, n (%)							0.47					0.02
Single	227	(3.2)	39	(4.2)	104	(3.9)		24	(2)	60	(2.6)	
Married	5322	(74.5)	780	(83.7)	2254	(85.3)		756	(61.5)	1532	(65.4)	
Widow/widower	1598	(22.4)	113	(12.1)	284	(10.7)		450	(36.6)	751	(32.1)	
Employment, n (%)							<0.001					0.12
Unemployed	3725	(52.1)	395	(42.4)	945	(35.8)		842	(68.5)	1543	(65.9)	
Employed	3422	(47.9)	537	(57.6)	1697	(64.2)		388	(31.5)	800	(34.1)	
Urbanization, n (%)							<0.001					0.40
Urban	3798	(53.1)	457	(49)	1485	(56.2)		627	(51)	1229	(52.5)	
Rural	3349	(46.9)	475	(51)	1157	(43.8)		603	(49)	1114	(47.5)	

n = number of participants; SD = standard deviation.

Continuous variable was performed by using Mann Whitney U test.

Categorical variables were performed by using chi-square test.

**Table 2.** Health, lifestyle behavior and sleep disturbance.

			Male (n = 3574)				*p*-value	Female (n = 3573)				*p*-value
	Total		Sleep Disturbance		No Sleep Disturbance			Sleep Disturbance		No Sleep Disturbance		
Variables	n	(%)	n	(%)	n	(%)		n	(%)	n	(%)	
	7,147	(100)	932	(26.1%)	2,642	(73.9%)		1,230	(34.4%)	2,343	(65.6%)	
Regular follow-up, n (%)							0.02					0.47
No	1,500	(21.0%)	228	(24.5%)	534	(20.2%)		268	(21.8%)	470	(20.1%)	
Sometime	2,826	(39.5%)	348	(37.3%)	1,037	(39.3%)		487	(39.6%)	954	(40.7%)	
Yes	2,821	(39.5%)	356	(38.2%)	1,071	(40.5%)		475	(38.6%)	919	(39.2%)	
Cancer, n (%)							0.04					0.61
No	7,099	(99.3%)	924	(99.1%)	2,633	(99.7%)		1,218	(99.0%)	2,324	(99.2%)	
Yes	48	(0.7%)	8	(0.9%)	9	(0.3%)		12	(1.0%)	19	(0.8%)	
Heart disease, n (%)							0.04					<0.001
No	6,447	(90.2%)	827	(88.7%)	2,403	(91.0%)		1,070	(87.0%)	2,147	(91.6%)	
Yes	700	(9.8%)	105	(11.3%)	239	(9.0%)		160	(13.0%)	196	(8.4%)	
Asthma, n (%)							0.03					<0.001
No	6,776	(94.8%)	871	(93.5%)	2,517	(95.3%)		1,143	(92.9%)	2,245	(95.8%)	
Yes	371	(5.2%)	61	(6.5%)	125	(4.7%)		87	(7.1%)	98	(4.2%)	
CKD, n (%)							0.19					0.43
No	7,010	(98.1%)	909	(97.5%)	2,595	(98.2%)		1,210	(98.4%)	2,296	(98.0%)	
Yes	137	(1.9%)	23	(2.5%)	47	(1.8%)		20	(1.6%)	47	(2.0%)	
Vigorous PA, n (%)							<0.01					0.95
No	6,295	(88.1%)	805	(86.4%)	2,177	(82.4%)		1,140	(92.7%)	2,173	(92.7%)	
Yes	852	(11.9%)	127	(13.6%)	465	(17.6%)		90	(7.3%)	170	(7.3%)	
Moderate PA, n (%)							<0.001					0.13
No	3,516	(49.2%)	587	(63.0%)	1,384	(52.4%)		553	(45.0%)	992	(42.3%)	
Yes	3,631	(50.8%)	345	(37.0%)	1,258	(47.6%)		677	(55.0%)	1,351	(57.7%)	
Alcohol consumption, n (%)							0.47					0.76 ^†^
No	7,014	(98.1%)	897	(96.2%)	2,556	(96.7%)		1,227	(99.8%)	2,334	(99.6%)	
Yes	133	(1.9%)	35	(3.8%)	86	(3.3%)		3	(0.2%)	9	(0.4%)	

n = number of participants; SD = standard deviation; CKD = chronic kidney disease; PA = physical activity.

Continuous variable was performed by using Mann Whitney U test.

Categorical variables were performed by using chi-square test or

^†^ Fisher’s exact test.

### 3.2 Prevalence of sleep disturbance

**Figure 2.**
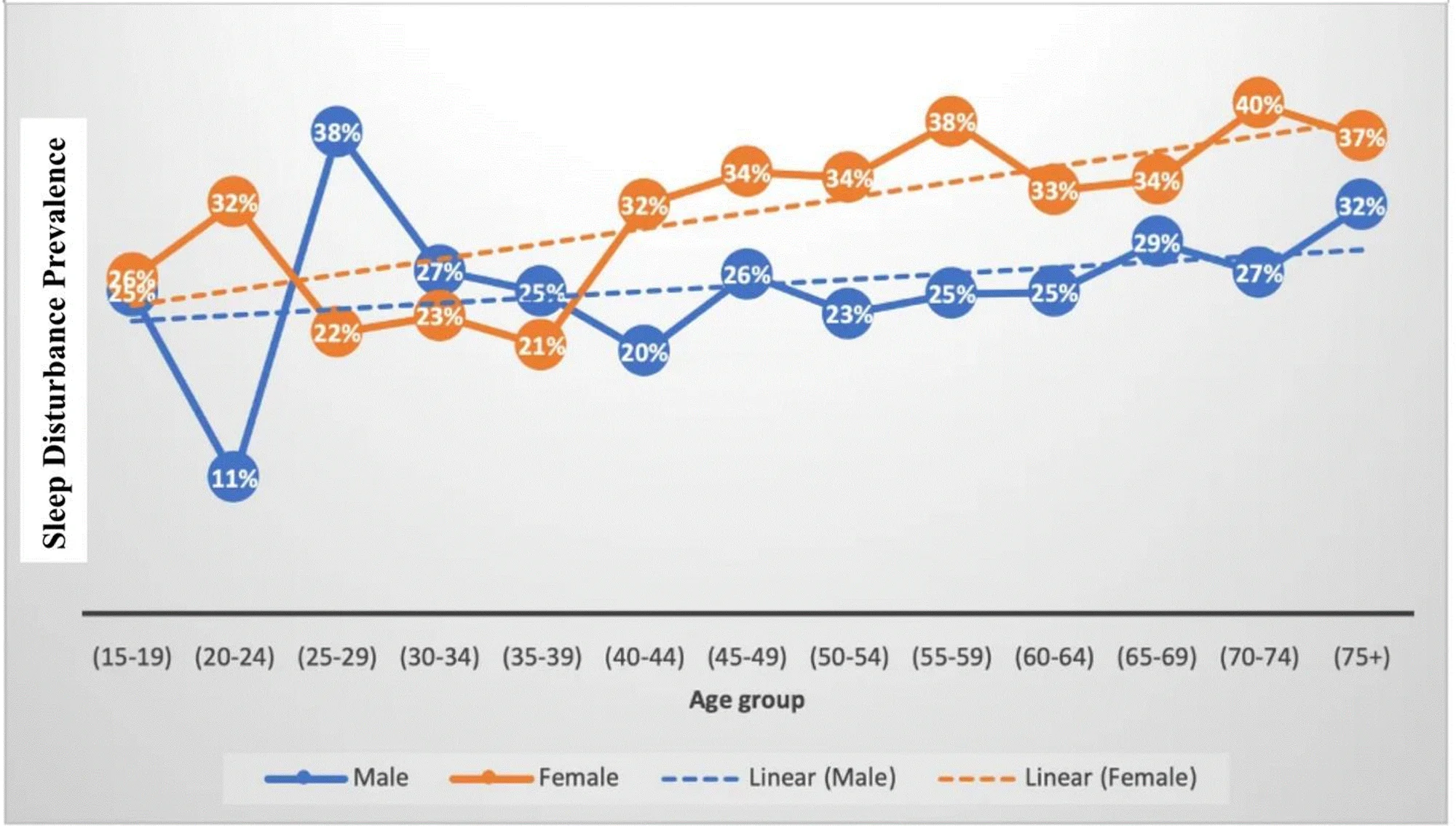
Sleep disturbance prevalence among stroke survivors by gender and age group.

The overall prevalence of sleep disturbance among stroke survivors in Indonesia was 28.4% (95% CI: 27.4%–29.4%). Figure 2 depicts the comparative prevalence of sleep disturbance among males and females across different age groups. Our findings suggest a general pattern of increasing prevalence with age, which was confirmed by a formal Cochran-Armitage test for trend (p < 0.01). We grouped the age into 13 age groups, ranging from 15–19 years old to more than 75 years old. There is a trend of increasing prevalence in the male sleep disturbance group, ranging from 25% to 32%. Similarly, we also find an increasing trend in sleep disturbance prevalence for the female age group (26% to 37%). Overall, we found that females have a higher trend in sleep disturbance prevalence compared to males (34.4% vs 26.1%, respectively). Age-specific denominators and 95% confidence intervals are provided in the supplementary materials.

### 3.3 Analysis of regression

The univariate regression analyses are displayed in Table 3 and Table 4. Multivariate regression analyses are displayed in Table 5 and Table 6. In the multivariate models, active smoking elevates the probability of sleep disturbance rate in comparison to non-smokers among subjects. Individuals with a greater educational attainment (university level) are less prone to have sleep disturbance. Individuals residing in urban environments are less prone to sleeplessness than those inhabiting rural regions. Individuals who partake in physical activity are less prone to have sleep disturbance. Female participants with comorbidities, including heart disease and asthma, exhibited a higher propensity for developing sleep disturbance. Formal interaction tests (sex × independent variable) revealed significant interactions for heart disease (p < 0.001) and asthma (p = 0.01), indicating that the associations of these comorbidities with sleep disturbance differed significantly between males and females.

**Table 3.** Associations between demographic characteristics and sleep disturbance using univariate logistic regression.

Variables	Male			Female		
	OR ^C^	(95% CI)	*P-value*	OR ^C^	(95% CI)	*P-value*
Age	1.008	(1.002 to 1.015)	<0.01	1.009	(1.003 to 1.014)	<0.01
Smoking status						
Non-smoker	Ref.			Ref.		
Yes (not every day)	1.216	(0.98 to 1.51)	0.08	0.898	(0.593 to 1.358)	0.61
Yes (everyday)	1.216	(1.027 to 1.441)	0.02	1.040	(0.684 to 1.579)	0.86
Education						
Non-university level	Ref.			Ref.		
No formal education	1.291	(0.976 to 1.708)	0.07	1.13	(0.926 to 1.38)	0.23
University level	0.601	(0.456 to 0.848)	<0.001	0.683	(0.496 to 0.942)	0.02
Marital status						
Widow/widower	Ref.			Ref.		
Single	0.942	(0.614 to 1.446)	0.79	0.668	(0.41 to 1.087)	0.07
Married	0.87	(0.689 to 1.098)	0.24	0.824	(0.712 to 0.953)	<0.01
Employment						
Employed	Ref.			Ref.		
Unemployed	1.321	(1.134 to 1.538)	<0.001	1.125	(0.971 to 1.304)	0.12
Urbanization						
Rural	Ref.			Ref.		
Urban	0.75	(0.645 to 0.871)	<0.001	0.943	(0.821 to 1.082)	0.4

OR ^C^ = crude odds ratio of univariate model; CI = confidence interval; Ref. = reference category.

**Table 4.** Association between health, lifestyle behavior and sleep disturbance using univariate logistic regression.

Variables	Male			Female		
	OR ^C^	(95% CI)	P-value	OR ^C^	(95% CI)	P-value
Regular follow-up						
No	Ref.			Ref.		
Sometime	0.786	(0.645 to 0.957)	0.02	0.895	(0.744 to 1.078)	0.24
Yes	0.779	(0.64 to 0.947)	0.01	0.906	(0.752 to 1.092)	0.3
Cancer						
No	Ref.			Ref.		
Yes	2.533	(0.974 to 6.584)	0.06	1.205	(0.583 to 2.491)	0.62
Heart disease						
No	Ref.			Ref.		
Yes	1.277	(1.001 to 1.627)	0.04	1.638	(1.313 to 2.044)	<0.001
Asthma						
No	Ref.			Ref.		
Yes	1.41	(1.028 to 1.934)	0.03	1.744	(1.295 to 2.348)	<0.001
CKD						
No	Ref.			Ref.		
Yes	1.397	(0.844 to 2.314)	0.19	0.807	(0.476 to 1.369)	0.43
Vigorous PA						
No	Ref.			Ref.		
Yes	0.739	(0.597 to 0.913)	<0.01	1.009	(0.774 to 1.316)	0.95
Moderate PA						
No	Ref.			Ref.		
Yes	0.647	(0.555 to 0.754)	<0.001	0.899	(0.782 to 1.033)	0.13
Alcohol consumption						
No	Ref.			Ref.		
Yes	1.16	(0.777 to 1.73)	0.47	0.634	(0.171 to 2.346)	0.49

OR ^C^ = crude odds ratio of univariate model; CI = confidence interval; Ref. = reference category; CKD = chronic kidney disease; PA = physical activity.

**Table 5.** Associations between demographic characteristics and sleep disturbance using logistic regression.

Variables	Male			Female		
	OR ^A^	(95% CI)	*P-value*	OR ^A^	(95% CI)	*P-value*
Age	1.006	(1.000 to 1.013)	0.55	1.006	(1 to 1.013)	0.07
Smoking status						
Non-smoker	Ref.			Ref.		
Yes (not every day)	1.231	(0.989 to 1.533)	0.06	0.981	(0.642 to 1.499)	0.93
Yes (everyday)	1.259	(1.057 to 1.500)	0.01	0.841	(0.553 to 1.279)	0.42
Education						
Non-university level	Ref.			Ref.		
No formal education	1.125	(0.843 to 1.503)	0.42	1.027	(0.833 to 1.266)	0.8
University level	0.649	(0.493 to 0.854)	<0.01	0.720	(0.519 to 1.000)	0.05
Marital status						
Widow/widower	Ref.			Ref.		
Single	1.127	(0.706 to 1.800)	0.62	0.826	(0.495 to 1.377)	0.46
Married	0.984	(0.772 to 1.254)	0.89	0.912	(0.773 to 1.074)	0.27
Employment						
Employed	Ref.			Ref.		
Unemployed	1.094	(0.919 to 1.302)	0.31	1.037	(0.885 to 1.214)	0.65
Urbanization						
Rural	Ref.			Ref.		
Urban	0.767	(0.656 to 0.896)	<0.01	0.934	(0.810 to 1.077)	0.34

OR ^A^ = adjusted odds ratio of multivariate model; CI = confidence interval; Ref. = reference category.

Adjusted model includes all variables presented in the table simultaneously using forced entry method. Hosmer-Lemeshow goodness-of-fit test: male (χ ^2^ = 8.42, p = 0.39), female (χ ^2^ = 9.15, p = 0.33).

**Table 6.** Association between health, lifestyle behavior and sleep disturbance using multivariable logistic regression.

Variables	Male			Female		
	OR ^A^	(95% CI)	*P-value*	OR ^A^	(95% CI)	*P-value*
Regular follow-up						
No	Ref.			Ref.		
Sometime	0.816	(0.667 to 0.997)	0.04	0.906	(0.751 to 1.092)	0.29
Yes	0.833	(0.68 to 1.021)	0.07	0.916	(0.758 to 1.107)	0.36
Cancer						
No	Ref.			Ref.		
Yes	2.483	(0.935 to 6.589)	0.07	1.104	(0.53 to 2.301)	0.79
Heart disease						
No	Ref.			Ref.		
Yes	1.282	(0.997 to 1.648)	0.05	1.615	(1.287 to 2.025)	<0.001
Asthma						
No	Ref.			Ref.		
Yes	1.297	(0.937 to 1.795)	0.12	1.658	(1.226 to 2.243)	<0.01
CKD						
No	Ref.			Ref.		
Yes	1.375	(0.819 to 2.309)	0.23	0.724	(0.422 to 1.241)	0.24
Vigorous PA						
No	Ref.			Ref.		
Yes	0.736	(0.583 to 0.929)	0.01	1.076	(0.817 to 1.418)	0.6
Moderate PA						
No	Ref.			Ref.		
Yes	0.679	(0.577 to 0.800)	<0.001	0.987	(0.847 to 1.150)	0.87
Alcohol consumption						
No	Ref.			Ref.		
Yes	1.334	(0.876 to 2.032)	0.18	0.61	(0.162 to 2.288)	0.46

OR ^A^ = adjusted odds ratio of multivariate model; CI = confidence interval; Ref. = reference category; CKD = chronic kidney disease; PA = physical activity.

Adjusted model includes all variables presented in the table simultaneously using forced entry method. Hosmer-Lemeshow goodness-of-fit test: male (χ ^2^ = 7.89, p = 0.44), female (χ ^2^ = 8.56, p = 0.38).

## 4. Discussion

This study is the first to investigate the impact of gender on the prevalence of sleep disturbance among stroke survivors in Indonesia, to the best of our knowledge. Our findings indicate that the overall prevalence of sleep disturbance remains high among the Indonesian population. It is worth noting that females have a higher prevalence of sleep disturbance than males. Our research should be considered credible due to the utilization of a substantial sample size and stringent methodology. However, the cross-sectional design of the RISKESDAS data does not allow us to confirm that the sleep disturbance occurred after the stroke. We therefore refer to our outcome as “sleep disturbance among stroke survivors” rather than “post-stroke sleep disturbance.”

It is important to observe that the prevalence of sleep disturbance following a stroke has increased over time. Females were found to have a higher increase in prevalence than males. Consistent with a previous study, increasing age was associated with the presence of sleep disturbance. ^11^ Moreover, females exhibited a greater prevalence of sleep disturbances than males among American adults. ^12^ This indicates the necessity of improving sleep instruction uniformly across various genders.

The study reveals a prevalence of sleep disturbance at 28.4%. This contrasts with previous meta-analyses that suggested the prevalence of sleep disturbance was between 35% and 43%. ^1^ ^,^ ^2^ The disparity in sleep disturbance prevalence arises from the variability in assessment methodologies; research employing standardized diagnoses reveal lower prevalence rates than those utilizing self-reported instruments. ^2^ ^,^ ^13^ However, this present study has a higher estimation compared to previous work that used hospital-based data and found the sleep disturbance incidence to be 8.2% in Taiwan (using ICD codes). ^4^ Early detection of sleep disturbance using precise measurement scales is critical.

Although the mechanics underlying sleep disturbance remain undetermined, the putative mechanisms can be explained in part. The stroke’s site, including the right hemisphere, thalamus, or brainstem, is associated with sleep disturbance. ^14^ Moreover, a confluence of environmental factors (e.g., hospitalization) ^15^ and biological factors (e.g., heightened sympathetic activation, intermittent hypoxemia, oxidative stress, inflammatory processes leading to frequent arousals, and disrupted sleep-wake regulation) may exacerbate post-stroke sleep disturbance. ^16^ ^,^ ^17^

Interestingly, male participants engage in physical activity associated with fewer sleep disturbance symptoms. A previous meta-analysis found that exercise may increase sleep quality. ^18^ The results are also consistent with previous research indicating that exercise improves sleep quality across all age groups. ^19^ ^–^ ^22^ Hence, we recommend regular exercise to improve sleep quality.

This study identifies a number of limitations. First, as noted above, the single-item measure of sleep disturbance does not fulfill clinical diagnostic criteria for insomnia. Our findings represent self-reported sleep disturbance rather than clinically diagnosed insomnia. Second, the cross-sectional design precludes establishing temporality or causality; we cannot confirm that the sleep disturbance began after the stroke. Third, the analysis did not incorporate the complex survey design features (weights, clustering, stratification) of RISKESDAS, limiting the generalizability of our estimates. Fourth, the failure to acquire pertinent confounders, including dietary details, environmental influences, and possible hypnotic usage, may compromise internal validity. Fifth, missing data were handled using complete-case analysis, which may introduce bias. Sixth, while we observed higher prevalence in females (34.4%) than males (26.1%), we did not perform a formal statistical test for this difference, as our primary objective was to identify sex-specific correlates. Future studies using design-based analyses with survey weights are needed to formally evaluate this difference. Age-standardized comparisons were also beyond our scope. Despite these limitations, this study possesses several strengths. The study population was selected from volunteers across Indonesia, assuring broad representation. Secondly, each interviewer received training to comprehend the structure and methodology of the questionnaire.

## 5. Conclusions

In conclusion, the prevalence of sleep disturbance following stroke remains high over time among stroke survivors in Indonesia. Females have a higher prevalence of sleep disturbance compared to males. These preliminary findings provide crucial insights for healthcare providers and policymakers. Implementing early sleep disturbance screening with accurate metrics is strongly advised. Future studies should employ validated insomnia assessment tools and longitudinal designs to establish the temporal relationship between stroke and sleep disturbance.

## Ethics statement

Ethical approval for the original RISKESDAS 2018 data collection was granted by the Ethical Committee of Health Research, NIHRD, Ministry of Health, Republic of Indonesia (No. LB.02.01/2/KE.267/2017). Secondary analysis for the present study was approved by the Health Research Ethics Committee of the Faculty of Medicine, Universitas Halu Oleo (No. 62/UN29.20.2/ETIK/2025).

## Informed consent statement

Written informed consent was obtained from all participants (or their legal guardians for minors or cognitively impaired individuals) during the original RISKESDAS 2018 data collection. As the present study involves only secondary analysis of de-identified, publicly available data, no additional informed consent was required.

## Data Availability

All datasets supporting this article are accessible via the following link: https://doi.org/10.5281/zenodo.20229933. ^23^ Data are available under the terms of the Creative Commons Attribution 4.0 International. Zenodo: Supplementary data: https://doi.org/10.5281/zenodo.20229933. ^23^ This project contains the following extended data:
• Tripod Checklist.docx. Data are available under the terms of the Creative Commons Attribution 4.0 International.
