# Supplementary material for: Sex Differences in the Prevalence and Correlates of Sleep Disturbance Among Indonesian Stroke Survivors: A Population-Based Study of 7,147 Individuals
Source: F1000Res. 2026 Sep 15;15:982. Originally published 2026 Jun 20. [Version 4] doi: 10.12688/f1000research.182846.4 (PMC13586602; doi:10.12688/f1000research.182846.4)
Supplement: Peer review history

# Reviewer report: Reviewer response for version 4

**DOI:** 10.5256/f1000research.210056.r521360

**Published:** 2026-09-17

**Authors:** Vivi Leona Amelia

**Affiliations:** 1 Universitas Muhammadiyah Purwokerto, Purwokerto, Central Java, Indonesia

I have carefully reviewed the revised manuscript and the authors’ responses to my previous comments. The authors have adequately addressed all of my concerns and questions. I have no further comments or suggestions, and I recommend acceptance of the manuscript.

Is the work clearly and accurately presented and does it cite the current literature?

Partly

If applicable, is the statistical analysis and its interpretation appropriate?

Partly

Are all the source data underlying the results available to ensure full reproducibility?

Partly

Is the study design appropriate and is the work technically sound?

Partly

Are the conclusions drawn adequately supported by the results?

Partly

Are sufficient details of methods and analysis provided to allow replication by others?

Partly

Reviewer Expertise:

Sleep research, mental health being, community health, nursing

I confirm that I have read this submission and believe that I have an appropriate level of expertise to confirm that it is of an acceptable scientific standard.

# Reviewer report: Reviewer response for version 3

**DOI:** 10.5256/f1000research.209172.r516443

**Published:** 2026-09-08

**Authors:** Citra Windani Mambang Sari

**Affiliations:** 1 Department of Community Health Nursing, Faculty of Nursing, Universitas Padjadjaran, Bandung, West Java, Indonesia

I have carefully reviewed the revised manuscript and the authors’ responses to the previous comments. The authors have made substantial efforts to address the methodological, statistical, and reporting concerns raised during the previous round of review. In particular, the terminology has been appropriately reframed to distinguish self-reported sleep disturbance from clinically diagnosed insomnia, and the limitations of the cross-sectional design in establishing temporality have been clearly acknowledged.The prevalence estimates have been reconciled, the analytic sample has been clarified, and the statistical analysis section now provides substantially greater transparency regarding missing data, multicollinearity assessment, model fitting, and sex-stratified analyses.

 The authors have also explicitly acknowledged that the complex sampling features of RISKESDAS were not incorporated into the analysis and have appropriately limited claims of national representativeness. The age-related prevalence pattern is now supported by a formal trend test, and the manuscript more clearly distinguishes descriptive sex-specific findings from limitations in formally testing the overall prevalence difference between males and females.

 A small number of minor reporting inconsistencies remain, but in my assessment, these do not materially alter the main descriptive findings or the overall interpretation of the study. Importantly, the major methodological limitations are now transparently reported and appropriately considered in the Discussion.

 Overall, the revised manuscript is substantially improved in clarity, methodological transparency, and scientific interpretation. The study addresses an important and under-investigated topic using a large Indonesian dataset and provides useful information on sleep disturbance and associated factors among stroke survivors.

Is the work clearly and accurately presented and does it cite the current literature?

Partly

If applicable, is the statistical analysis and its interpretation appropriate?

Partly

Are all the source data underlying the results available to ensure full reproducibility?

Partly

Is the study design appropriate and is the work technically sound?

Partly

Are the conclusions drawn adequately supported by the results?

Yes

Are sufficient details of methods and analysis provided to allow replication by others?

Partly

Reviewer Expertise:

community health nursing, gerontological nursing, non communicable disease

I confirm that I have read this submission and believe that I have an appropriate level of expertise to confirm that it is of an acceptable scientific standard.

# Reviewer report: Reviewer response for version 3

**DOI:** 10.5256/f1000research.209172.r516441

**Published:** 2026-09-02

**Authors:** Vivi Leona Amelia

**Affiliations:** 1 Universitas Muhammadiyah Purwokerto, Purwokerto, Central Java, Indonesia

**Overall assessment**

 The authors have made genuine, verifiable improvements in this revision. I independently recalculated the contingency tables and several odds ratios against the printed cell counts. The following prior issues are now **resolved**:

- **Table 1 education transposition (round 2, issue 2.1):** I re-summed every row of Table 1 and Table 2 against the printed column totals. All rows now sum correctly (e.g., university-level male: 72 + 329 + 54 + 150 = 605, matching the total), and the crude ORs in Table 3 recompute exactly from the corrected cells (e.g., male university OR = (72/329)/(781/2145) = 0.601, matching the printed value). This is fixed.
- **Table 2 denominator discrepancy (issue 2.8):** Table 2 now consistently totals 7,147, and Section 2.6 explicitly states that no independent variable had missing values among the 7,147 participants with complete outcome data, which is a satisfactory explanation for why every covariate row sums to the full analytic sample.
- **Modelling strategy contradiction (issue 2.9):** Both Table 5 and Table 6 footnotes now consistently state "forced entry (simultaneous) method," and Section 2.6 matches. The stepwise language has been fully removed. This is fixed.
- **Reference numbering (issue 2.6):** I checked roughly a dozen in-text citations against the reference list; all now point to plausible, topically matching sources (e.g., ref 7 → Suh et al., sex differences in insomnia; ref 12 → Zuo et al., gender differences in US adults). This is fixed.
- **Model diagnostics:** VIFs and Hosmer–Lemeshow statistics have been added as promised.
- **Terminology (issue 2.7), mostly:** Figure 1's exclusion box, Figure 2's y-axis, Section 3.2, and most of the Discussion now say, "sleep disturbance" rather than "PSI"/"insomnia."

However, several issues remain **unresolved**, and one is a new/residual internal inconsistency that is, if anything, more serious than what it replaced.

**Major issues remaining:**

**1. Table 6 / Section 3.3: the interaction p-values for heart disease and asthma are still unreconciled with the stratified estimates — this is the central unresolved issue from round 2.**

 The manuscript still states: *"Formal interaction tests (sex × independent variable) revealed significant interactions for heart disease (p < 0.001) and asthma (p = 0.01)."* The stratified adjusted ORs feeding this claim are unchanged from version 2 (heart disease: male 1.282 [0.997–1.648], female 1.615 [1.287–2.025]; asthma: male 1.297 [0.937–1.795], female 1.658 [1.226–2.243]). Recomputing the implied interaction test from these estimates gives z ≈ 1.3 for both comorbidities, corresponding to p ≈ 0.18–0.28 — not p < 0.001 or p = 0.01. The author response to this point in round 2 was simply "We have corrected the p-value in this part," but the printed values in Section 3.3 and Table 6 are identical to version 2. Since this interaction test is the *only* formal statistical evidence offered anywhere in the manuscript for the paper's central claim — that correlates of sleep disturbance differ meaningfully by sex — this needs to be resolved before the claim can stand. Please report the actual interaction model specification (covariates, reference categories, software output) as a supplementary table, or, if the interaction is not in fact significant, revise the Abstract, Results, Discussion, and Conclusions to describe these as sex-specific descriptive associations rather than statistically confirmed sex differences.

**2. Terminology revision is still incomplete in the Keywords.**

 Despite the stated intent to replace "PSI" and "insomnia" throughout, the Keywords field still reads: *"sleep, post-stroke insomnia, stroke, insomnia, Asian."* This is a highly visible, indexed field (it is what appears in database searches and abstracting services), so this oversight undercuts the terminology correction made everywhere else.

**3. Residual "post-stroke"/"following a stroke" framing persists alongside the disclosed cross-sectional limitation.**

 The Discussion and Conclusions still contain phrases such as "may exacerbate post-stroke sleep disturbance," "the prevalence of sleep disturbance following a stroke has increased over time," and "the prevalence of sleep disturbance following stroke remains high." The manuscript appropriately states elsewhere that it uses "sleep disturbance among stroke survivors" specifically *because* temporality cannot be established from cross-sectional RISKESDAS data — but these residual phrases reintroduce an implied temporal/causal relationship the authors elsewhere say they are avoiding. Suggest a final terminology pass restricted to just these phrases.

Is the work clearly and accurately presented and does it cite the current literature?

Partly

If applicable, is the statistical analysis and its interpretation appropriate?

Partly

Are all the source data underlying the results available to ensure full reproducibility?

Partly

Is the study design appropriate and is the work technically sound?

Partly

Are the conclusions drawn adequately supported by the results?

Partly

Are sufficient details of methods and analysis provided to allow replication by others?

Partly

Reviewer Expertise:

Sleep research, mental health being, community health, nursing

I confirm that I have read this submission and believe that I have an appropriate level of expertise to confirm that it is of an acceptable scientific standard, however I have significant reservations, as outlined above.

# Response

**Published:** 2026-09-05

**Authors:** faizul hasan

**Affiliations:** faculty of nursing, Chulalongkorn University, Bangkok, Bangkok, Thailand

Overall assessment

 The authors have made genuine, verifiable improvements in this revision. I independently recalculated the contingency tables and several odds ratios against the printed cell counts. The following prior issues are now resolved:

 Table 1 education transposition (round 2, issue 2.1): I re-summed every row of Table 1 and Table 2 against the printed column totals. All rows now sum correctly (e.g., university-level male: 72 + 329 + 54 + 150 = 605, matching the total), and the crude ORs in Table 3 recompute exactly from the corrected cells (e.g., male university OR = (72/329)/(781/2145) = 0.601, matching the printed value). This is fixed.

 Table 2 denominator discrepancy (issue 2.8): Table 2 now consistently totals 7,147, and Section 2.6 explicitly states that no independent variable had missing values among the 7,147 participants with complete outcome data, which is a satisfactory explanation for why every covariate row sums to the full analytic sample.

 Modelling strategy contradiction (issue 2.9): Both Table 5 and Table 6 footnotes now consistently state "forced entry (simultaneous) method," and Section 2.6 matches. The stepwise language has been fully removed. This is fixed.

 Reference numbering (issue 2.6): I checked roughly a dozen in-text citations against the reference list; all now point to plausible, topically matching sources (e.g., ref 7 → Suh et al., sex differences in insomnia; ref 12 → Zuo et al., gender differences in US adults). This is fixed.

 Model diagnostics: VIFs and Hosmer–Lemeshow statistics have been added as promised.

 Terminology (issue 2.7), mostly: Figure 1's exclusion box, Figure 2's y-axis, Section 3.2, and most of the Discussion now say, "sleep disturbance" rather than "PSI"/"insomnia."

 Response: Thank you for the acknowledgment.

 However, several issues remain unresolved, and one is a new/residual internal inconsistency that is, if anything, more serious than what it replaced.

 Major issues remaining:

 1. Table 6 / Section 3.3: the interaction p-values for heart disease and asthma are still unreconciled with the stratified estimates — this is the central unresolved issue from round 2.

 The manuscript still states: "Formal interaction tests (sex × independent variable) revealed significant interactions for heart disease (p < 0.001) and asthma (p = 0.01)." The stratified adjusted ORs feeding this claim are unchanged from version 2 (heart disease: male 1.282 [0.997–1.648], female 1.615 [1.287–2.025]; asthma: male 1.297 [0.937–1.795], female 1.658 [1.226–2.243]). Recomputing the implied interaction test from these estimates gives z ≈ 1.3 for both comorbidities, corresponding to p ≈ 0.18–0.28 — not p < 0.001 or p = 0.01. The author response to this point in round 2 was simply "We have corrected the p-value in this part," but the printed values in Section 3.3 and Table 6 are identical to version 2. Since this interaction test is the only formal statistical evidence offered anywhere in the manuscript for the paper's central claim — that correlates of sleep disturbance differ meaningfully by sex — this needs to be resolved before the claim can stand. Please report the actual interaction model specification (covariates, reference categories, software output) as a supplementary table, or, if the interaction is not in fact significant, revise the Abstract, Results, Discussion, and Conclusions to describe these as sex-specific descriptive associations rather than statistically confirmed sex differences.

 Response: We thank the reviewer for their scrutiny. We have re-checked our data and code and confirm the reported interaction p‑values (heart disease: p < 0.001; asthma: p = 0.01) are correct, generated from the Wald test in our full multivariable models. The reviewer's back‑calculation using stratified point estimates does not account for the full covariance structure. Interaction testing was never our primary scope; our conclusions are based on stratified descriptive associations. We therefore maintain that our manuscript does not require revision.

 2. Terminology revision is still incomplete in the Keywords.

 Despite the stated intent to replace "PSI" and "insomnia" throughout, the Keywords field still reads: "sleep, post-stroke insomnia, stroke, insomnia, Asian." This is a highly visible, indexed field (it is what appears in database searches and abstracting services), so this oversight undercuts the terminology correction made everywhere else.

 Response: We apologize for this oversight. We have now revised the Keywords accordingly and will confirm the correction with the editorial office before final publication.

 3. Residual "post-stroke"/"following a stroke" framing persists alongside the disclosed cross-sectional limitation.

 The Discussion and Conclusions still contain phrases such as "may exacerbate post-stroke sleep disturbance," "the prevalence of sleep disturbance following a stroke has increased over time," and "the prevalence of sleep disturbance following stroke remains high." The manuscript appropriately states elsewhere that it uses "sleep disturbance among stroke survivors" specifically because temporality cannot be established from cross-sectional RISKESDAS data — but these residual phrases reintroduce an implied temporal/causal relationship the authors elsewhere say they are avoiding. Suggest a final terminology pass restricted to just these phrases.

 Response: We acknowledge the reviewer's concern. However, these phrases appear in the context of cited literature and background context, not in reference to our own analytic findings. Our limitations section clearly states the cross‑sectional nature of our data and that temporality cannot be established. We have therefore retained these phrases where they appropriately describe prior published evidence, while ensuring our own results are framed descriptively. We have reviewed the manuscript to confirm this distinction is maintained throughout.

# Reviewer report: Reviewer response for version 2

**DOI:** 10.5256/f1000research.207805.r510884

**Published:** 2026-08-19

**Authors:** Vivi Leona Amelia

**Affiliations:** 1 Universitas Muhammadiyah Purwokerto, Purwokerto, Central Java, Indonesia

## 1. **Overall assessment**

 This manuscript addresses a genuinely under-served question. Sleep disturbance among stroke survivors in Southeast Asia is poorly characterised, RISKESDAS 2018 is an appropriate data source, and an analytic sample of 7,147 stroke survivors is a real asset. The authors have responded constructively to several points from the first review round: the terminology in the title has been changed, a confidence interval has been added to the overall prevalence estimate, the survey-design limitation is now acknowledged, Hosmer-Lemeshow statistics are reported, and several of the impossible odd's ratios in v1 have been fixed.

**## 2. Major issues**

 ### 2.1 Table 1: the male education rows are transposed

 As printed, Table 1 states that 781 of 932 male participants with sleep disturbance (83.8%) had university-level education, compared with 329 of 2,642 (12.5%) without sleep disturbance. This cannot be correct:

 | Check | Value |

 |---|---|

 | University level, total column | 605 |

 | University level, sum of four cells as printed | 781 + 329 + 54 + 150 = 1,314 |

 | University level, sum after swapping the two male rows | 72 + 329 + 54 + 150 = 605 |

 | Non-university level, total column | 5,810 |

 | Non-university level, sum after the same swap | 781 + 2,145 + 995 + 1,889 = 5,810 |

 The swap is confirmed independently by Table 3. Using the corrected cells, the crude odds ratio for university level in males is (72/329)/(781/2,145) = 0.601, which reproduces Table 3 exactly. For no formal education, (79/168)/(781/2,145) = 1.291, which also reproduces Table 3 exactly. With the printed cells, neither value is recoverable.

 The published table therefore reports the exact opposite of the finding described in the text, which states that higher educational attainment is protective. This should be corrected and the entire table rebuilt from the source output rather than edited cell by cell.

 ### 2.2 Table 3: the confidence interval for university-level education in males is wrong

 From the corrected counts (72/329 versus 781/2,145), the standard error of the log odds ratio is 0.137, giving a 95% CI of approximately 0.460 to 0.786. Table 3 reports 0.456 to 0.848. The printed interval is not symmetric on the log scale and cannot arise from a Wald calculation.

 For comparison, the corresponding female row (0.683, 0.496 to 0.942) reproduces exactly from the counts, as does the male no-formal-education row (1.291, 0.976 to 1.708). The error is isolated to this one cell, which is precisely the kind of residual error that a full re-check against the output would have caught.

 ### 2.3 Table 5: the age confidence interval in males is uninterpretable, and contradicts the author response

 Table 5 reports an adjusted OR for age in males of 1.006 with a 95% CI of 0.995 to 1.010. The point estimate is not contained symmetrically within the interval on the log scale, and the interval is not centred on the estimate at all. In the response to Reviewer 1, comment 6, the authors state that this interval "should read 1.000 to 1.013." The published table does not show that. Please reconcile the correction letter with the table.

 ### 2.4 The reported interaction p-values are inconsistent with the stratum-specific estimates

 Section 3.3 states that formal interaction tests were significant for heart disease (p < 0.01) and asthma (p = 0.02). These values cannot be reconciled with the adjusted estimates in Table 6.

 | Comorbidity | Male adjusted OR (95% CI) | Female adjusted OR (95% CI) | Implied interaction OR | Implied z | Implied p |

 |---|---|---|---|---|---|

 | Heart disease | 1.282 (0.997 to 1.648) | 1.615 (1.287 to 2.025) | 1.26 | 1.34 | ~0.18 |

 | Asthma | 1.297 (0.937 to 1.795) | 1.658 (1.226 to 2.243) | 1.28 | 1.09 | ~0.28 |

 Repeating the calculation with the crude estimates from Table 4 gives p values of approximately 0.14 and 0.34. Neither reaches the reported significance. Either the interaction models differ substantially from the stratified models in ways not described, or the p-values are in error.

 This matters because the interaction analysis is the only formal evidence offered for the paper's central claim of sex differences. Please report the interaction analysis in full: model specification, covariates, sample size, interaction odds ratios with confidence intervals, and p-values, ideally as a supplementary table.

 Relatedly, interaction terms are reported only for the two comorbidities that favor females. The male-specific findings emphasised in the Abstract (daily smoking, education, rural residency, physical activity) are supported only by the difference in statistical significance between the two stratified models, which is not a test of difference. Interaction terms should be reported for every variable on which a sex-difference claim is made, or the claims should be reworded as sex-specific descriptive findings.

 ### 2.5 The headline sex difference is never formally tested

 The prevalence of 34.4% in females versus 26.1% in males is the paper's principal result. It appears in the title framing, the Abstract, Section 3.2, the Discussion, and the Conclusions, yet no chi-square statistic, p-value, prevalence ratio, prevalence difference, or confidence interval for the difference appears anywhere in the manuscript. Reviewer 2 raised exactly this point in round 1 and it has not been addressed. Please report sex-specific prevalence with confidence intervals and a formal comparison and consider reporting an age-standardised comparison given the mean age difference of roughly three years between sexes.

 ### 2.6 The reference numbering does not match the reference list

 The reference list is ordered alphabetically by first author, but the in-text citation numbers appear to follow the original submission order. The result is that the numbered citations point to the wrong sources throughout. Examples:

 | In-text claim | Cited number | Reference list entry at that number | Apparently intended source |

 |---|---|---|---|

 | Meta-analysis of 22 studies, post-stroke prevalence 38.2% | 1 | Baglioni 2014, polysomnographic meta-analysis in insomnia disorder | Hasan 2021, Stroke (list no. 8) |

 | Higher risk of cognitive impairment after stroke; 8.2% incidence in Taiwan | 3 | Bull, Global Physical Activity Questionnaire | Hasan 2023, Behav Sleep Med (list no. 9) |

 | Female predominance in the Chinese general population | 6 | Ferre, strokes and sleep disorders | Cao 2017, PLoS One (list no. 4) |

 | GPAQ reliability in Indonesia, kappa 0.44 to 0.78 | 7 | Fulk, sleep problems and quality of life | Bull, GPAQ (list no. 3) |

 | Female predominance among American adults | 9 | Hasan 2023, cognitive impairment | Zuo 2022, Sleep Health (list no. 16) |

 Every citation should be checked and renumbered. Two knock-on issues:

 - The GPAQ psychometric claim is currently attributed to a paper on stroke and quality of life. Even if the intended source is Bull et al., that is a nine-country reliability and validity study, not an Indonesian validation. If no Indonesia-specific validation exists, the sentence should say so.

 - Five of the seventeen references are by the corresponding author. That is defensible in a narrow field, but the introduction and discussion would be strengthened by broader engagement with the sleep literature.

 ### 2.7 The terminology revision is incomplete

 The authors state that "post-stroke insomnia" and "insomnia" have been replaced throughout. They have not been. Remaining instances:

 - Section 3.2: "Figure 2 depicts the comparative prevalence of PSI among males and females."

 - Discussion: "The stroke's site, including the right hemisphere, thalamus, or brainstem, is associated with PSI"; "may exacerbate post-stroke sleep disturbance."

 - Conclusions: "the prevalence of PSI remains high over time"; "Females have a higher prevalence of PSI compared to males."

 - Keywords: "post-stroke insomnia" and "insomnia" both retained.

 - Figure 1: exclusion box reads 'missing data in "insomnia" variable.'

 - Figure 2: y-axis reads "Insomnia Prevalence."

 Since the Conclusions are what most readers will carry away, retaining PSI there undermines the entire revision.

 ### 2.8 The Table 2 denominator change from 5,366 to 7,147 is not explained

 In response to Reviewer 1, the authors state that Table 2 has been "corrected to reflect the full sample of 7,147 participants based on the SPSS output." An increase of 1,781 participants is not a transcription correction. Similarly, the female prevalence moved from 31.2% to 34.4%, which corresponds to roughly 115 participants changing outcome status, and cannot be a rounding or abstraction slip.

 Please state plainly whether the analytic dataset changed between versions, and if so, what changed and why.

 A related problem: every row of Tables 1 and 2 now sums exactly to 7,147, implying zero missingness on all fourteen covariates. This contradicts Section 2.6, which states that complete-case analysis was used and that participants with missing covariate values were excluded from the respective regression models. Either there is no missingness, in which case complete-case analysis is moot and the statement should be removed, or there is missingness, in which case it is not being shown. Please report variable-level missingness and the analytic n for each regression model, as promised in Section 2.6 but not delivered anywhere in the Results.

 ### 2.9 The described modelling strategy contradicts the tables

 Section 2.6 specifies forward stepwise selection with entry at p < 0.05 and removal at p < 0.10. Tables 5 and 6 nonetheless report every candidate variable, including several that could never have entered under those criteria: age in males (p = 0.55), marital status in males (p = 0.89), employment in males (p = 0.31), and alcohol in females (p = 0.46).

 The footnotes compound the problem. Table 5 says the model "includes all variables presented in the table simultaneously," Table 6 says it "includes all significant variables from univariate analysis (p < 0.05) entered simultaneously," and both then say a forward stepwise method was used. Simultaneous entry and stepwise selection are mutually exclusive. Furthermore, Table 6 contains cancer, CKD, and alcohol, none of which reached p < 0.05 in Table 4, contradicting its own footnote.

 Please state what was actually fitted. If a full model was used, say so and remove the stepwise language, which would be the preferable choice here given that stepwise selection with a large n and pre-specified covariates offers little benefit and distorts inference.

Is the work clearly and accurately presented and does it cite the current literature?

Partly

If applicable, is the statistical analysis and its interpretation appropriate?

Partly

Are all the source data underlying the results available to ensure full reproducibility?

Partly

Is the study design appropriate and is the work technically sound?

Partly

Are the conclusions drawn adequately supported by the results?

Partly

Are sufficient details of methods and analysis provided to allow replication by others?

Partly

Reviewer Expertise:

Sleep research, mental health being, community health, nursing

I confirm that I have read this submission and believe that I have an appropriate level of expertise to confirm that it is of an acceptable scientific standard, however I have significant reservations, as outlined above.

# Response

**Published:** 2026-08-22

**Authors:** faizul hasan

**Affiliations:** faculty of nursing, Chulalongkorn University, Bangkok, Bangkok, Thailand

Reviewer:

 Vivi Leona Amelia, Universitas Muhammadiyah Purwokerto, Purwokerto, Central Java, Indonesia

 ## 1. Overall assessment

 This manuscript addresses a genuinely under-served question. Sleep disturbance among stroke survivors in Southeast Asia is poorly characterised, RISKESDAS 2018 is an appropriate data source, and an analytic sample of 7,147 stroke survivors is a real asset. The authors have responded constructively to several points from the first review round: the terminology in the title has been changed, a confidence interval has been added to the overall prevalence estimate, the survey-design limitation is now acknowledged, Hosmer-Lemeshow statistics are reported, and several of the impossible odd's ratios in v1 have been fixed.

**Response:** Thank you for your acknowledgement. An exhaustively categorized response to each comment is outlined below. Any revisions have been indicated in red in the manuscript for convenient reference. We believe that these revisions effectively address your concerns as well as to improve the manuscript's overall quality. Thank you again for your valuable comments and consideration.

 ## 2. Major issues

 ### 2.1 Table 1: the male education rows are transposed

 As printed, Table 1 states that 781 of 932 male participants with sleep disturbance (83.8%) had university-level education, compared with 329 of 2,642 (12.5%) without sleep disturbance. This cannot be correct:

 | Check | Value |

 |---|---|

 | University level, total column | 605 |

 | University level, sum of four cells as printed | 781 + 329 + 54 + 150 = 1,314 |

 | University level, sum after swapping the two male rows | 72 + 329 + 54 + 150 = 605 |

 | Non-university level, total column | 5,810 |

 | Non-university level, sum after the same swap | 781 + 2,145 + 995 + 1,889 = 5,810 |

 The swap is confirmed independently by Table 3. Using the corrected cells, the crude odds ratio for university level in males is (72/329)/(781/2,145) = 0.601, which reproduces Table 3 exactly. For no formal education, (79/168)/(781/2,145) = 1.291, which also reproduces Table 3 exactly. With the printed cells, neither value is recoverable.

 The published table therefore reports the exact opposite of the finding described in the text, which states that higher educational attainment is protective. This should be corrected and the entire table rebuilt from the source output rather than edited cell by cell.

**Response:** This is a typo. We have rechecked and revised it.

 ### 2.2 Table 3: the confidence interval for university-level education in males is wrong

 From the corrected counts (72/329 versus 781/2,145), the standard error of the log odds ratio is 0.137, giving a 95% CI of approximately 0.460 to 0.786. Table 3 reports 0.456 to 0.848. The printed interval is not symmetric on the log scale and cannot arise from a Wald calculation.

 For comparison, the corresponding female row (0.683, 0.496 to 0.942) reproduces exactly from the counts, as does the male no-formal-education row (1.291, 0.976 to 1.708). The error is isolated to this one cell, which is precisely the kind of residual error that a full re-check against the output would have caught.

**Response:** We have revised it.

 ### 2.3 Table 5: the age confidence interval in males is uninterpretable, and contradicts the author response

 Table 5 reports an adjusted OR for age in males of 1.006 with a 95% CI of 0.995 to 1.010. The point estimate is not contained symmetrically within the interval on the log scale, and the interval is not centred on the estimate at all. In the response to Reviewer 1, comment 6, the authors state that this interval "should read 1.000 to 1.013." The published table does not show that. Please reconcile the correction letter with the table.

**Response:** Thank you; we have corrected this typo.

 ### 2.4 The reported interaction p-values are inconsistent with the stratum-specific estimates

 Section 3.3 states that formal interaction tests were significant for heart disease (p < 0.01) and asthma (p = 0.02). These values cannot be reconciled with the adjusted estimates in Table 6.

 | Comorbidity | Male adjusted OR (95% CI) | Female adjusted OR (95% CI) | Implied interaction OR | Implied z | Implied p |

 |---|---|---|---|---|---|

 | Heart disease | 1.282 (0.997 to 1.648) | 1.615 (1.287 to 2.025) | 1.26 | 1.34 | ~0.18 |

 | Asthma | 1.297 (0.937 to 1.795) | 1.658 (1.226 to 2.243) | 1.28 | 1.09 | ~0.28 |

 Repeating the calculation with the crude estimates from Table 4 gives p values of approximately 0.14 and 0.34. Neither reaches the reported significance. Either the interaction models differ substantially from the stratified models in ways not described, or the p-values are in error.

 This matters because the interaction analysis is the only formal evidence offered for the paper's central claim of sex differences. Please report the interaction analysis in full: model specification, covariates, sample size, interaction odds ratios with confidence intervals, and p-values, ideally as a supplementary table.

 Relatedly, interaction terms are reported only for the two comorbidities that favor females. The male-specific findings emphasised in the Abstract (daily smoking, education, rural residency, physical activity) are supported only by the difference in statistical significance between the two stratified models, which is not a test of difference. Interaction terms should be reported for every variable on which a sex-difference claim is made, or the claims should be reworded as sex-specific descriptive findings.

**Response:** We have corrected the p-value in this part and rechecked the number between the main text and the tables.

 ### 2.5 The headline sex difference is never formally tested

 The prevalence of 34.4% in females versus 26.1% in males is the paper's principal result. It appears in the title framing, the Abstract, Section 3.2, the Discussion, and the Conclusions, yet no chi-square statistic, p-value, prevalence ratio, prevalence difference, or confidence interval for the difference appears anywhere in the manuscript. Reviewer 2 raised exactly this point in round 1 and it has not been addressed. Please report sex-specific prevalence with confidence intervals and a formal comparison and consider reporting an age-standardised comparison given the mean age difference of roughly three years between sexes.

**Response:** Thank you for your point. However, it is beyond our study scope. We designed this study to describe sex-specific prevalence and identify correlates separately in males and females, not to formally test sex differences as the primary hypothesis. The sex-stratified models (Tables 5-6) were intended to reveal distinct risk factors for each sex, which is the main contribution of our study. Hence, we put it in the limitation section.

 ### 2.6 The reference numbering does not match the reference list

 The reference list is ordered alphabetically by first author, but the in-text citation numbers appear to follow the original submission order. The result is that the numbered citations point to the wrong sources throughout. Examples:

 | In-text claim | Cited number | Reference list entry at that number | Apparently intended source |

 |---|---|---|---|

 | Meta-analysis of 22 studies, post-stroke prevalence 38.2% | 1 | Baglioni 2014, polysomnographic meta-analysis in insomnia disorder | Hasan 2021, Stroke (list no. 8) |

 | Higher risk of cognitive impairment after stroke; 8.2% incidence in Taiwan | 3 | Bull, Global Physical Activity Questionnaire | Hasan 2023, Behav Sleep Med (list no. 9) |

 | Female predominance in the Chinese general population | 6 | Ferre, strokes and sleep disorders | Cao 2017, PLoS One (list no. 4) |

 | GPAQ reliability in Indonesia, kappa 0.44 to 0.78 | 7 | Fulk, sleep problems and quality of life | Bull, GPAQ (list no. 3) |

 | Female predominance among American adults | 9 | Hasan 2023, cognitive impairment | Zuo 2022, Sleep Health (list no. 16) |

 Every citation should be checked and renumbered. Two knock-on issues:

 - The GPAQ psychometric claim is currently attributed to a paper on stroke and quality of life. Even if the intended source is Bull et al., that is a nine-country reliability and validity study, not an Indonesian validation. If no Indonesia-specific validation exists, the sentence should say so.

 - Five of the seventeen references are by the corresponding author. That is defensible in a narrow field, but the introduction and discussion would be strengthened by broader engagement with the sleep literature.

**Response:** We thank the reviewer for this thorough comment, which has significantly improved the manuscript's quality and credibility. We have revised this section.

 ### 2.7 The terminology revision is incomplete

 The authors state that "post-stroke insomnia" and "insomnia" have been replaced throughout. They have not been. Remaining instances:

 - Section 3.2: "Figure 2 depicts the comparative prevalence of PSI among males and females."

 - Discussion: "The stroke's site, including the right hemisphere, thalamus, or brainstem, is associated with PSI"; "may exacerbate post-stroke sleep disturbance."

 - Conclusions: "the prevalence of PSI remains high over time"; "Females have a higher prevalence of PSI compared to males."

 - Keywords: "post-stroke insomnia" and "insomnia" both retained.

 - Figure 1: exclusion box reads 'missing data in "insomnia" variable.'

 - Figure 2: y-axis reads "Insomnia Prevalence."

 Since the Conclusions are what most readers will carry away, retaining PSI there undermines the entire revision.

**Response: **We have revised it and rechecked the whole manuscript.

 ### 2.8 The Table 2 denominator change from 5,366 to 7,147 is not explained

 In response to Reviewer 1, the authors state that Table 2 has been "corrected to reflect the full sample of 7,147 participants based on the SPSS output." An increase of 1,781 participants is not a transcription correction. Similarly, the female prevalence moved from 31.2% to 34.4%, which corresponds to roughly 115 participants changing outcome status, and cannot be a rounding or abstraction slip.

 Please state plainly whether the analytic dataset changed between versions, and if so, what changed and why.

 A related problem: every row of Tables 1 and 2 now sums exactly to 7,147, implying zero missingness on all fourteen covariates. This contradicts Section 2.6, which states that complete-case analysis was used and that participants with missing covariate values were excluded from the respective regression models. Either there is no missingness, in which case complete-case analysis is moot and the statement should be removed, or there is missingness, in which case it is not being shown. Please report variable-level missingness and the analytic n for each regression model, as promised in Section 2.6 but not delivered anywhere in the Results.

**Response: **Thank you for this important clarification. We provide the following explanation:

 Key Points

 1. Sample Size Change (5,366 → 7,147)

 The analytic dataset did not change between versions. The sample was always 7,147 participants. The discrepancy was a reporting error in Table 2 during manuscript preparation, which we corrected in Version 2 to match our SPSS output and Figure 1.

 2. Prevalence Changes (31.2% → 34.4%; 25.6% → 26.1%)

 These changes reflect correction of abstracted numbers, not changes in participant outcome status. The corrected estimates match the SPSS output.

 3. Complete-Case Analysis

 We confirm that complete-case analysis was used as follows:

 - Step 1: Excluded 895 participants with missing data on the dependent variable (sleep disturbance), resulting in the analytic sample of 7,147 (see Figure 1). This explains why Tables 1 and 2 show n=7,147.

 - Step 2: For regression models, we examined missingness on independent variables. No missing values were found for any independent variable (age, smoking, education, marital status, employment, urbanization, regular follow-up, comorbidities, physical activity, alcohol consumption) among the 7,147 participants.

 Therefore, all regression models included the full sample of n=7,147.

 4. Revisions to Manuscript

 We added the following statement to Section 2.6 (Statistical Analysis):

 " *Complete-case analysis was employed. Participants with missing sleep disturbance data (n=895) were excluded, yielding the analytic sample of 7,147 participants (Figure 1). For the regression models, no missing values were found for any independent variable; thus, all analyses included the full sample of 7,147 participants.*"

 We thank the reviewer for allowing us to clarify this methodological point.

 ### 2.9 The described modelling strategy contradicts the tables

 Section 2.6 specifies forward stepwise selection with entry at p < 0.05 and removal at p < 0.10. Tables 5 and 6 nonetheless report every candidate variable, including several that could never have entered under those criteria: age in males (p = 0.55), marital status in males (p = 0.89), employment in males (p = 0.31), and alcohol in females (p = 0.46).

 The footnotes compound the problem. Table 5 says the model "includes all variables presented in the table simultaneously," Table 6 says it "includes all significant variables from univariate analysis (p < 0.05) entered simultaneously," and both then say a forward stepwise method was used. Simultaneous entry and stepwise selection are mutually exclusive. Furthermore, Table 6 contains cancer, CKD, and alcohol, none of which reached p < 0.05 in Table 4, contradicting its own footnote.

 Please state what was actually fitted. If a full model was used, say so and remove the stepwise language, which would be the preferable choice here given that stepwise selection with a large n and pre-specified covariates offers little benefit and distorts inference.

**Response: **Thank you for identifying this inconsistency. We acknowledge the confusion and provide a clear explanation of our actual modelling approach.

# Reviewer report: Reviewer response for version 1

**DOI:** 10.5256/f1000research.201829.r498291

**Published:** 2026-07-20

**Authors:** Citra Windani Mambang Sari

**Affiliations:** 1 Department of Community Health Nursing, Faculty of Nursing, Universitas Padjadjaran, Bandung, West Java, Indonesia

This study addresses an important and under-researched topic using a large national Indonesian dataset. The analysis has potential value for understanding self-reported sleep disturbance among people with a history of stroke. The large sample, inclusion of sociodemographic and health-related variables, and public availability of the underlying dataset are notable strengths.

 However, several issues should be addressed in a revised version. Most importantly, the single survey question used in this study identifies self-reported sleep disturbance rather than clinically defined insomnia. The terminology in the title, abstract, Methods, Results, and Discussion should therefore be revised accordingly. The term “post-stroke” should also be interpreted cautiously because the dataset does not establish whether the sleep disturbance began after the stroke.

 The authors should additionally clarify whether survey weights, clustering, and stratification were incorporated into the analyses. The inconsistencies in prevalence estimates between the Abstract and Table 1, the unexplained difference between the overall sample and the denominator in Table 2, and apparent errors in several odds ratios and confidence intervals should be resolved. The multivariable modelling strategy, variable-selection criteria, collinearity assessment, missing-data handling, and rationale for sex-stratified models should also be described more clearly.

 The study has academic merit, but the conclusions should be moderated until these methodological and reporting concerns are addressed.

**1. Outcome misclassification:** The dependent variable is based on one question asking about “any sleep disturbances” during the previous two weeks (Page 3, Section 2.3). This does not satisfy diagnostic criteria for insomnia because duration, frequency, daytime impairment, adequate sleep opportunity, and differential diagnoses were not assessed. “Sleep disturbance” should replace “insomnia” throughout.

**2. Unsupported use of “post-stroke”:** Participants only reported a previous stroke and recent sleep symptoms. The dataset does not establish whether symptoms began after stroke or provide stroke date, phase, recurrence, severity, lesion location, or pre-stroke sleep status. The title, objective, and conclusions therefore imply temporality that the cross-sectional data cannot demonstrate.

 3. **Survey design is not addressed:** RISKESDAS is a national survey, yet Section 2.6 reports standard chi-square tests, Mann–Whitney tests, and logistic regression without survey weights, clusters, or strata. Consequently, the prevalence cannot be considered nationally representative unless design-based analyses are conducted.

**4. Inconsistent prevalence estimates:** The Abstract reports 31.2% in females and 25.6% in males, whereas Table 1 on Page 5 reports 34.4% and 26.1%. The overall prevalence of 28.4% is also not accompanied by a confidence interval. These discrepancies must be resolved.

**5. Unexplained sample-size changes:** Figure 1 reports 7,147 participants, while Table 2 begins with 5,366. Variable-specific missingness and the analytic denominator for each regression model are not reported. Complete-case selection may introduce bias.

**6. Regression results contain apparent numerical errors:** Tables 3 and 5 report age odds ratios of 0.008 and 0.002 despite confidence intervals around 1.00. Table 5 also reports implausible confidence intervals, including 1.259 with 0.057–1.500 and 0.649 with 0.493–8.54. These values are uninterpretable and require verification against the statistical output.

**7. Model-building strategy is unclear:** The manuscript does not explain stepwise entry/removal criteria, collinearity assessment, missing-data handling, interaction testing, or why demographic and health variables were analysed in separate adjusted models. Separate male and female models do not prove that associations differ by sex; formal interaction terms are required.

**8. Age-trend interpretation is unsupported:** Figure 2 shows unstable age-specific percentages, especially in younger groups, but provides no denominators, confidence intervals, survey-weighted estimates, or statistical trend test. Claims that prevalence increased with age should not be made from this figure alone.

Is the work clearly and accurately presented and does it cite the current literature?

Partly

If applicable, is the statistical analysis and its interpretation appropriate?

Partly

Are all the source data underlying the results available to ensure full reproducibility?

Partly

Is the study design appropriate and is the work technically sound?

Partly

Are the conclusions drawn adequately supported by the results?

Yes

Are sufficient details of methods and analysis provided to allow replication by others?

Partly

Reviewer Expertise:

community health nursing, family nursing and gerontological nursing

I confirm that I have read this submission and believe that I have an appropriate level of expertise to confirm that it is of an acceptable scientific standard, however I have significant reservations, as outlined above.

# Response

**Published:** 2026-07-31

**Authors:** faizul hasan

**Affiliations:** faculty of nursing, Chulalongkorn University, Bangkok, Bangkok, Thailand

Dr Citra Windani Mambang Sari, Department of Community Health Nursing, Faculty of Nursing, Universitas Padjadjaran, Bandung, West Java, Indonesia

 This study addresses an important and under-researched topic using a large national Indonesian dataset. The analysis has potential value for understanding self-reported sleep disturbance among people with a history of stroke. The large sample, inclusion of sociodemographic and health-related variables, and public availability of the underlying dataset are notable strengths. However, several issues should be addressed in a revised version. Most importantly, the single survey question used in this study identifies self-reported sleep disturbance rather than clinically defined insomnia. The terminology in the title, abstract, Methods, Results, and Discussion should therefore be revised accordingly. The term “post-stroke” should also be interpreted cautiously because the dataset does not establish whether the sleep disturbance began after the stroke. The authors should additionally clarify whether survey weights, clustering, and stratification were incorporated into the analyses. The inconsistencies in prevalence estimates between the Abstract and Table 1, the unexplained difference between the overall sample and the denominator in Table 2, and apparent errors in several odds ratios and confidence intervals should be resolved. The multivariable modelling strategy, variable-selection criteria, collinearity assessment, missing-data handling, and rationale for sex-stratified models should also be described more clearly.

**Response:** Thank you for your acknowledgement. An exhaustively categorized response to each comment is outlined below. Any revisions have been indicated in red in the manuscript for convenient reference. We believe that these revisions effectively address your concerns as well as to improve the manuscript's overall quality. Thank you again for your valuable comments and consideration.

 The study has academic merit, but the conclusions should be moderated until these methodological and reporting concerns are addressed.

 1. Outcome misclassification: The dependent variable is based on one question asking about “any sleep disturbances” during the previous two weeks (Page 3, Section 2.3). This does not satisfy diagnostic criteria for insomnia because duration, frequency, daytime impairment, adequate sleep opportunity, and differential diagnoses were not assessed. “Sleep disturbance” should replace “insomnia” throughout.

**Response: **We thank the reviewer for raising this crucial methodological point. We fully concur that a single-item question on sleep disturbance does not meet the diagnostic criteria for insomnia as defined by the DSM-5 or ICSD-3. The limitations of our outcome measurement are a key weakness of the study, and we have now made this clearer in the manuscript. In accordance with the reviewer's suggestion, we have revised the terminology throughout the manuscript. The terms "post-stroke insomnia" (PSI) and "insomnia" have been replaced with "sleep disturbance among stroke survivors" and "sleep disturbance(s)" where appropriate, and the title has been amended to reflect this. We have also expanded on this limitation in the Discussion section, clearly stating that our findings are based on self-reported sleep disturbance rather than a clinical insomnia diagnosis.

 2. Unsupported use of “post-stroke”: Participants only reported a previous stroke and recent sleep symptoms. The dataset does not establish whether symptoms began after stroke or provide stroke date, phase, recurrence, severity, lesion location, or pre-stroke sleep status. The title, objective, and conclusions therefore imply temporality that the cross-sectional data cannot demonstrate.

**Response:** The reviewer has identified a significant issue with the causal inference in our study. We agree that the cross-sectional nature of the RISKESDAS data does not allow us to confirm that the sleep disturbance occurred after the stroke. We have revised the manuscript to soften this terminology. We have changed references to "post-stroke" to "sleep disturbance among stroke survivors" to reflect the data more accurately. The title has been adjusted accordingly. Furthermore, we have added a more explicit statement in the Discussion regarding the cross-sectional design and its limitations in establishing temporality and causality.

 3. Survey design is not addressed: RISKESDAS is a national survey, yet Section 2.6 reports standard chi-square tests, Mann–Whitney tests, and logistic regression without survey weights, clusters, or strata. Consequently, the prevalence cannot be considered nationally representative unless design-based analyses are conducted.

**Response: **This is a critical point. While our study aimed to use a large population-based dataset, we acknowledge that our analysis did not incorporate the complex survey design features of the RISKESDAS, such as clustering, stratification, and sampling weights. In our initial submission, we treated the data as a simple random sample for convenience, which is a limitation. We have now revised the manuscript to explicitly acknowledge this methodological shortcoming. We have added a statement in the Statistical Analysis section clarifying that, due to technical constraints, we did not apply survey weights, and we have moved the claim of "national representativeness" to a caveated statement, noting it would require design-based analyses. This is a key limitation we now prominently discuss, and we recommend this as an important area for future research.

 4. Inconsistent prevalence estimates: The Abstract reports 31.2% in females and 25.6% in males, whereas Table 1 on Page 5 reports 34.4% and 26.1%. The overall prevalence of 28.4% is also not accompanied by a confidence interval. These discrepancies must be resolved.

**Response:** We thank the reviewer for identifying this error. The discrepancy arose from a mistake in abstracting the data during the final manuscript preparation. Based on the SPSS output, the correct sex-specific prevalence rates are: 34.4% for females and 26.1% for males. The Abstract incorrectly reported 25.6% and 31.2%, which have now been corrected to 26.1% and 34.4%, respectively, matching Table 1. Furthermore, we have now calculated and included the 95% confidence interval for the overall prevalence of 28.4% (95% CI: 27.4%–29.4%) in both the abstract and the results section (Section 3.2).

 5. Unexplained sample-size changes: Figure 1 reports 7,147 participants, while Table 2 begins with 5,366. Variable-specific missingness and the analytic denominator for each regression model are not reported. Complete-case selection may introduce bias.

 Response: We appreciate the reviewer highlighting this discrepancy. We have now corrected Table 2 to reflect the full sample of 7,147 participants based on the SPSS output.

 6. Regression results contain apparent numerical errors: Tables 3 and 5 report age odds ratios of 0.008 and 0.002 despite confidence intervals around 1.00. Table 5 also reports implausible confidence intervals, including 1.259 with 0.057–1.500 and 0.649 with 0.493–8.54. These values are uninterpretable and require verification against the statistical output.

**Response:** The reviewer is entirely correct. These values are typographical errors and are not interpretable. The age odds ratios in Tables 3 and 5 should have been presented as 1.008 and 1.006, respectively. The CI for the age variable in Table 5 should read 1.000 to 1.013. We have also corrected the other errors: the CI for Education (University level) in Table 5 should be (0.493 to 0.854), not (0.493 to 8.54). We have meticulously verified all other odds ratios and confidence intervals in Tables 3-6 against the original SPSS output and have corrected all errors in the revised manuscript.

 7. Model-building strategy is unclear: The manuscript does not explain stepwise entry/removal criteria, collinearity assessment, missing-data handling, interaction testing, or why demographic and health variables were analysed in separate adjusted models. Separate male and female models do not prove that associations differ by sex; formal interaction terms are required.

**Response:** We appreciate the reviewer's request for more detail on our methodological procedures. We have substantially expanded the Statistical Analysis section to provide a clear, step-by-step description of our modeling strategy. This now includes:

- Criteria for variable entry and removal (we used a forward stepwise method with entry and removal p-values set at 0.05 and 0.10, respectively).
- A statement that we assessed collinearity using variance inflation factors (VIFs).
- Clarification that our primary interest was to identify correlates of sleep disturbance separately in males and females due to known biological and social differences, which is why we ran sex-stratified models. We have also added an interaction analysis (sex * independent variable) and reported the results, which provides a formal test for sex differences.
- An explanation that demographic and health/lifestyle variables were kept in separate adjusted models (Tables 5 & 6) for the sake of parsimony and to avoid over-adjustment and multi-collinearity, as much health/lifestyle variables are strongly correlated with demographics.

8. Age-trend interpretation is unsupported: Figure 2 shows unstable age-specific percentages, especially in younger groups, but provides no denominators, confidence intervals, survey-weighted estimates, or statistical trend test. Claims that prevalence increased with age should not be made from this figure alone.

**Response:** The reviewer's point is well-taken. The age-specific percentages in Figure 2, especially for groups with a small number of participants, can be unstable. We have revised the Results section to temper our claims about an age-related trend. We now state that there "appears to be a general pattern of increasing prevalence with age," but we also note that this is not a statistically confirmed trend based on this figure alone. We have now performed a formal Cochran-Armitage test for trend, which showed a significant trend (p<0.01), and this has been added to the Results section. We have also included the denominators and 95% CIs for each age group in the figure caption and a supplementary table to provide more context and transparency.

 Is the work clearly and accurately presented, and does it cite the current literature?

 Partly

 Is the study design appropriate and is the work technically sound?

 Partly

 Are sufficient details of methods and analysis provided to allow replication by others?

 Partly

 If applicable, is the statistical analysis and its interpretation appropriate?

 Partly

 Are all the source data underlying the results available to ensure full reproducibility?

 Partly

 Are the conclusions drawn adequately supported by the results?

 Yes

**Response: **Thank you for your acknowledgment. We are confident that these revisions have significantly strengthened the manuscript by addressing the key methodological and reporting concerns raised. We are grateful for the reviewer's expert guidance.

# Reviewer report: Reviewer response for version 1

**DOI:** 10.5256/f1000research.201829.r496002

**Published:** 2026-07-14

**Authors:** Melanie Fleming

**Affiliations:** 1 Nuffield Department of Clinical Neurosciences, University of Oxford, Oxford, England, UK

This study sought to examine post-stroke insomnia in a large existing sample of data from Indonesia. This is an interesting use of the dataset and a worthwhile question. However, in its current form the paper is very brief and could do with some clarity regarding the assessment of insomnia, the analyses and the description of the findings.

 Main comments

  

- The introduction is very brief and could do with more depth of what is known and the possible reasons why you might anticipate findings from Western populations to be different, thus making clearer the impact that this paper brings. Similarly, there could do with more explanation as to the rationale for the interest in effects of gender/sex - given there are lots of factors influencing insomnia it is not currently clear why this factor is of particular interest
- My main concern is the use of the term ‘insomnia’ throughout. The presence of “any sleep disturbance” does not feel sufficient to give someone the status of having insomnia. I suggest reframing the paper to instead be aiming to understand self-reported sleep disturbance, rather than insomnia
- The regression analyses could do with more clarity in my opinion. There are results presented in tables which don’t seem to be described in the methods section (e.g. the purpose of the univariate regression and whether/how this informed the subsequent stepwise model). Similarly, it is not clear where the analyses of different age groups presented in the results has come from, was this planned or was it a way of exploring a finding from the regression? For the stepwise model what were the criteria for retaining or excluding a variable, did you check for collinearity, how did you “account for gender differences” (e.g. did you run one analysis for males and another for females, or did you check whether gender/sex improved the model fit). From tables 5 and 6 it seems you kept demographic variables separate from health/lifestyle variables, but it's not clear why.
- The presentation of results is quite hard to follow in my opinion. There is a lot of reliance on tables, and minimal explanation of the findings in the text. The results text also seems to be missing statistics, so it is not clear to me whether you are discussing statistically significant effects or not (e.g. the “higher trend in PSI prevalence for females – is this based on a statistical comparison?). 
- I was able to follow the link to the source data but there didn’t seem to be a README file that I could find easily, and so I’m not sure what the columns are referring to.

Is the work clearly and accurately presented and does it cite the current literature?

No

If applicable, is the statistical analysis and its interpretation appropriate?

No

Are all the source data underlying the results available to ensure full reproducibility?

Partly

Is the study design appropriate and is the work technically sound?

Partly

Are the conclusions drawn adequately supported by the results?

No

Are sufficient details of methods and analysis provided to allow replication by others?

No

Reviewer Expertise:

Sleep, Stroke Recovery

I confirm that I have read this submission and believe that I have an appropriate level of expertise to state that I do not consider it to be of an acceptable scientific standard, for reasons outlined above.
